# Supplementary material for: DMRforPairs: identifying Differentially Methylated Regions between unique samples using array based methylation profiles
Source: BMC Bioinformatics. 2014 May 15;15:141. doi: 10.1186/1471-2105-15-141 (PMC4046028; doi:10.1186/1471-2105-15-141)
Supplement: Additional file 2 — DMRforPairs output for the comparison of A431-MCF7 and NA17018-NA17105. Please start from the HTML files in each folder. Available via the BMC Bioinformatics website. [file 1471-2105-15-141-S2.zip › 1394847754114233_MOESM2_ESM/A431_MCF7/figures/22346.pdf]

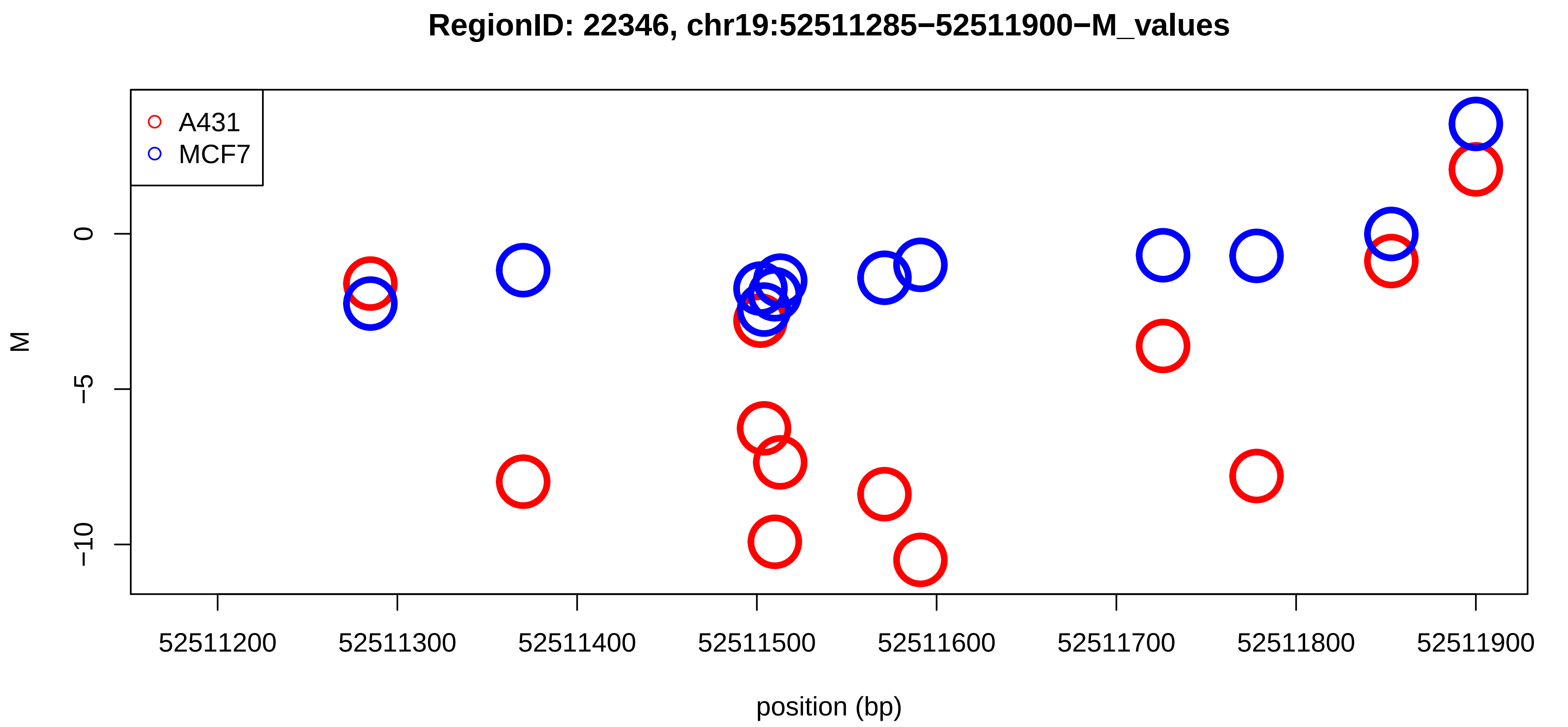
RegionID: 22346, chr19:52511285−52511900−M_values
A431
MCF7
M
0
−5
−10
52511200
52511300
52511400
52511500
52511600
52511700
52511800
52511900
position (bp)

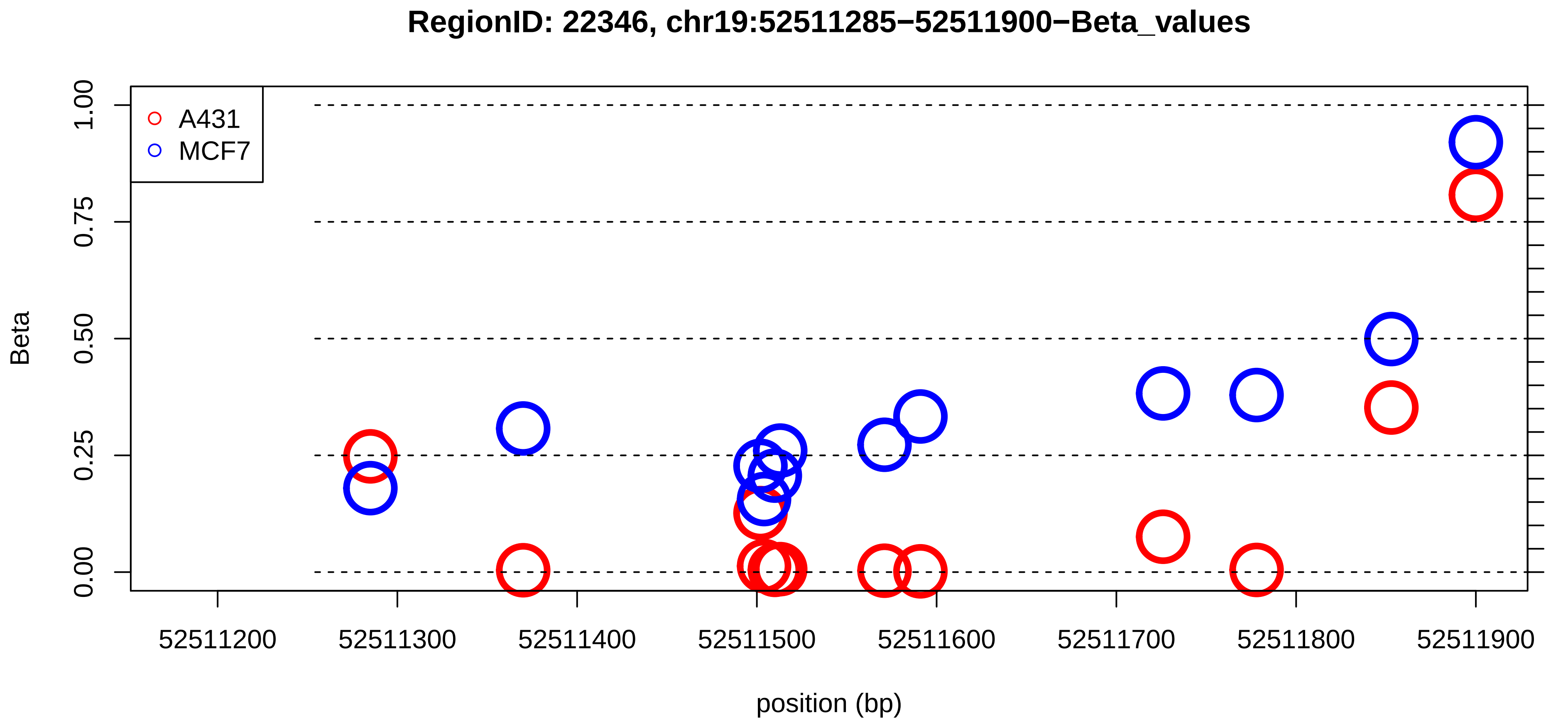
RegionID: 22346, chr19:52511285−52511900−Beta_values
A431
MCF7
Beta
1.00
0.75
0.50
0.25
0.00
52511200
52511300
52511400
52511500
52511600
52511700
52511800
52511900
position (bp)

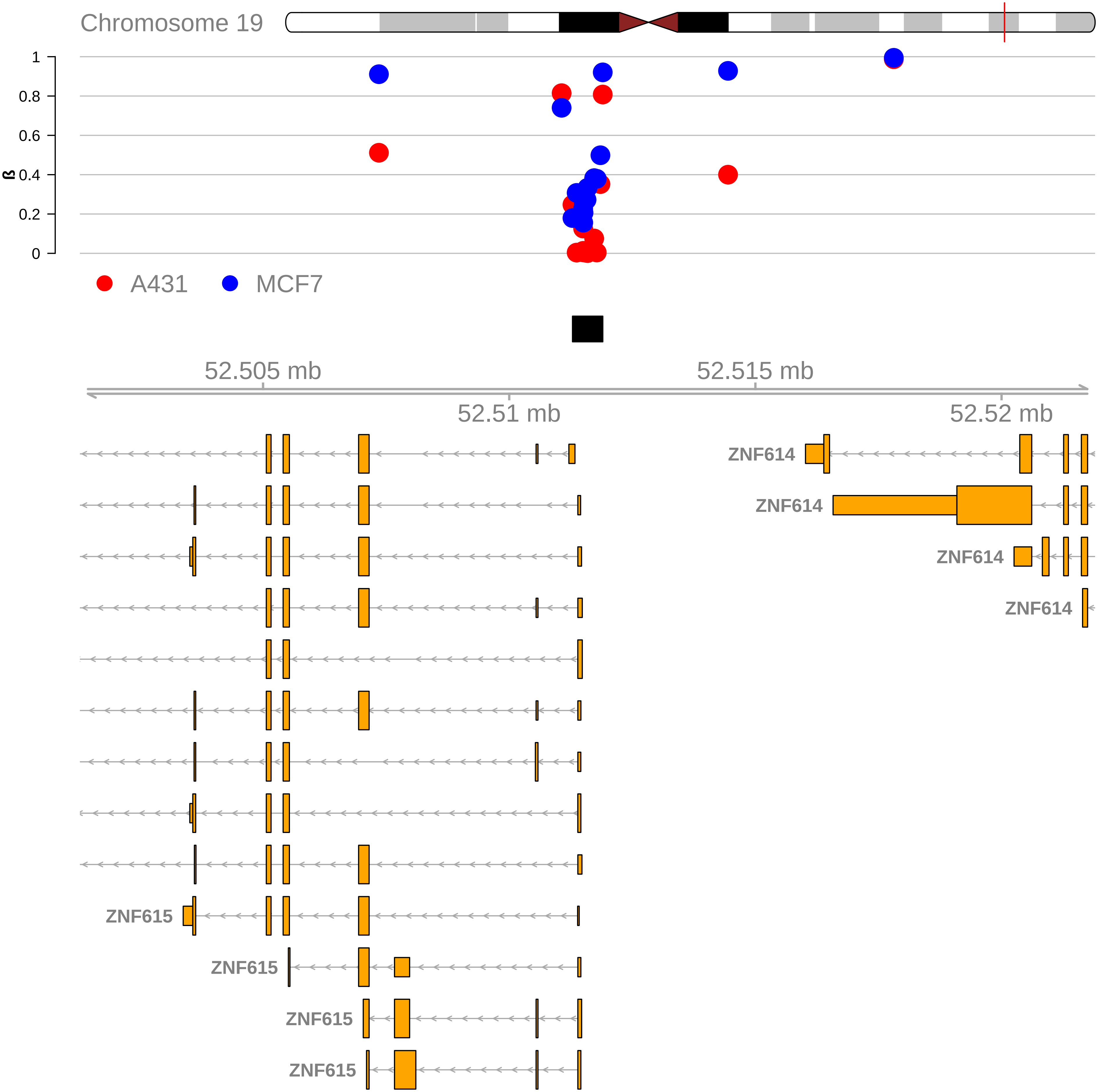
Chromosome 19
1
0.8
0.6
ß 0.4
0.2
0
A431
MCF7
52.505 mb
52.515 mb
52.51 mb
52.52 mb
ZNF614
ZNF614
ZNF614
ZNF614
ZNF615
ZNF615
ZNF615
ZNF615
